# Donor-Specific Regulatory T Cells Acquired from Tolerant Mice Bearing Cardiac Allograft Promote Mixed Chimerism and Prolong Intestinal Allograft Survival

**DOI:** 10.3389/fimmu.2016.00511

**Published:** 2016-11-17

**Authors:** Xiao-Fei Shen, Jin-Peng Jiang, Jian-Jun Yang, Wei-Zhong Wang, Wen-Xian Guan, Jun-Feng Du

**Affiliations:** ^1^Department of General Surgery, Affiliated Drum Tower Hospital of Nanjing University Medical School, Nanjing, China; ^2^Department of Rehabilitation Medicine, PLA Army General Hospital, Beijing, China; ^3^Division of Digestive Surgery, Xijing Hospital of Digestive Diseases, Fourth Military Medical University, Xi’an, China; ^4^Department of General Surgery, PLA Army General Hospital, Beijing, China

**Keywords:** donor-specific regulatory T cells, mixed chimerism, transplantation tolerance, small bowel transplantation, bone marrow transplantation

## Abstract

The induction of donor-specific transplant tolerance has always been a central problem for small bowel transplantation (SBT), which is thought to be the best therapy for end-stage bowel failure. With the development of new tolerance-inducing strategies, mixed chimerism induced by co-stimulation blockade has become most potent for tolerance of allografts, such as skin, kidney, and heart. However, a lack of clinically available co-stimulation blockers has hindered efficient application in humans. Furthermore, unlike those for other types of solid organ transplantation, strategies to induce robust mixed chimerism for intestinal allografts have not been fully developed. To improve current mixed chimerism induction protocols for future clinical application, we developed a new protocol using donor-specific regulatory T (Treg) cells from mice with heart allograft tolerance, immunosuppressive drugs which could be used clinically and low doses of irradiation. Our results demonstrated that donor-specific Treg cells acquired from tolerant mice after *in vitro* expansion generate stable chimerism and lead to acceptance of intestinal allograft. Increased intragraft Treg cells and clonal deletion contribute to the development of SBT tolerance.

## Introduction

For patients with end-stage bowel failure, small bowel transplantation (SBT) is recognized as a definitive therapy (1). However, the intestine carries the largest population of lymphoid cells of any transplanted solid organ, which are the least tolerogenic cells in any organ and they have the potential risk of inducing graft-versus-host reaction (2). Therefore, both acute and chronic rejection after SBT is still a great challenge to overcome, which leads to the inferior overall outcome of SBT when compared to that of other transplanted organs (3). Mixed hematopoietic chimerism, in which both donor and host stem cells contribute to hematopoiesis, could help to achieve potent donor-specific tolerance across full MHC barriers (4). Establishment of mixed chimerism through transplantation of donor bone marrow into recipients is one of the most promising strategies for inducing transplantation tolerance (5). Most of the chimerism-inducing protocols require the use of co-stimulation blockade agents (6). Recent results from experimental mouse studies based on co-stimulation blockade induction of stable mixed chimerism are encouraging. However, translation of tolerance protocols from preclinical animal studies to the clinic is still a major challenge due to the lack of clinically available co-stimulation blockers (6). Regulatory T (Treg) cells have long been recognized to play a critical role in self-tolerance, but administration of Treg cells on their own does not induce robust immune tolerance across MHC barriers in immunocompetent hosts (7). Combining Treg cell therapy with co-stimulation blockade and rapamycin has been tested to promote full MHC-mismatched mixed chimerism, and the results are encouraging (8, 9). Furthermore, recipient donor-specific Treg (DSTreg) cells are thought to be the most potent to promote mixed chimerism among all types of Treg cells (9). However, whether recipient DSTreg cells could lead to intestinal allograft acceptance after establishment of mixed chimerism has not been fully elucidated.

Our previous work (10) demonstrated that allograft acceptance can be established by donor-specific transfusion with complete blockade of inducible co-stimulator (ICOS)/B7h signaling. Furthermore, this allograft acceptance was transferable and maintained by CD4^+^CD25^+^ T cells from recipient mice with long-term allograft survival, and these Treg cells could be expanded *in vitro* and exert donor-specific immune negative regulation. In the present study, a non-myeloablative protocol of combined transfusion of DSTreg cells and donor bone marrow, together with cytotoxic T lymphocyte antigen CTLA4Ig (abatacept, clinically available co-stimulation blocker) and rapamycin, was developed to establish mixed chimerism in lightly irradiated mice. We evaluated the possibility of mixed chimerism to induce murine SBT tolerance and tried to develop new methods for clinical use.

## Materials and Methods

### Animals

Male mice of inbred strains BALB/c (H-2^d^), C57BL/6 (B6, H-2^b^), and C3H/HeJ (C3H, H-2^k^) aged 6–8 weeks were obtained from the Experimental Animal Center of the Fourth Military Medical University (Xi’an, China). All the animal experiments were carried out following the Guidelines for the Care and Use of Laboratory Animals of the Fourth Military Medical University and were approved by the ethical review committee of the Fourth Military Medical University.

### Bone Marrow Preparation and Treatment Regimens

Age-matched male mice received 3 Gy total body irradiation (Day −1), co-stimulation blockade with abatacept (0.5 mg/mouse, Day 2) (Orencia, Bristol-Myers Squibb Pharmaceuticals, Princeton, NJ, USA), and three doses of rapamycin (0.1mg/mouse, Days −1, 0, and 1) (LC Laboratories, Woburn, MA, USA) were injected intravenously on day 0 with 2.0 × 10^7^ unseparated bone marrow cells (BMCs) harvested from MHC-full mismatched BALB/c donors (8–12 weeks old), with or without expanded fresh Treg cells or expanded DSTreg cells (3 × 10^6^ per mouse). The preparation of BM of BALB/c mice was performed as previously described (11).

### SBT and Histological Graft Assessment

Heterotopic SBT was performed using a modified technique of Guo et al. (11). Briefly, about 5 cm of ileum was removed from donor mice on a vascular pedicle consisting of the superior mesenteric artery, abdominal aorta, and portal vein. The donor abdominal aorta was anastomosed end-to-side to the recipient infrarenal aorta and the donor portal vein to the recipient inferior vena cava. The proximal and distal ends of the intestinal graft were exteriorized stomas. Intestinal allografts were scored according to the following definitions: 0, no rejection; 1, scattered apoptotic crypt cells; 2, focal crypt destruction; and 3, mucosal ulceration with or without transmural necrosis (11).

### Skin Grafting

Full-thickness tail skin from donor (BALB/c) and fully mismatched third-party (C3H) mice was grafted 100 days after SBT and visually inspected at short intervals thereafter. Grafts were considered to be rejected when <10% remained viable (12).

### Isolation of CD4^+^CD25^+^ Treg Cells

CD4^+^CD25^+^ Treg cells were isolated as previously described (8). Fresh or DSTreg cells were isolated from spleen of naïve or tolerant B6 mice. CD4^+^CD25^+^ cells were purified by magnetic bead separation using negative selection for CD4^+^ and subsequent positive selection for CD25^+^ by incubating CD4^+^ enriched cells with phycoerythrin (PE)-conjugated α-CD25 (PC61) followed by α-PE microbeads (CD4^+^CD25^+^ Regulatory T-cell Isolation Kit; MiltenyiBiotec, Bergisch Gladbach, Germany) (13). The purity of separated cells was >90%.

### Generation of Dendritic Cells

Bone marrow-derived dendritic cells (BMDCs) were induced in the medium of 4 ml complete RPMI 1640, by adding 20 ng/ml granulocyte-macrophage colony-stimulating factor (GM-CSF). On days 3 and 5, the culture medium was replaced by fresh medium with GM-CSF (20 ng/ml). On day 6, cells were additionally treated with 1 μg/ml lipopolysaccharide for 24 h to further induce the maturation of DC. Loosely adherent cells and those in the culture supernatant were harvested by gentle washing with PBS for further use.

### Treg-Cell Proliferation Assay

Sorted CD4^+^CD8^−^CD25^+^ T cells (5 × 10^4^) from naïve and tolerant B6 mice bearing cardiac grafts were cultured for 14 days at 37°C in 5% CO_2_ with 2 × 10^5^ BALB/c BMDCs in the presence or absence of interleukin (IL)-2 (1000 U/ml) and rapamycin (100 nM), alone or in combination. In some experiments, T cells were prelabeled with a solution of 5 mM carboxyflurescein diacetate succinmidyl ester (CFSE; Invitrogen, Carlsbad, CA, USA), followed by culture for 7 days with various stimuli. CFSE dilution was analyzed on a Beckman Coulter Epics XL.

### CD4^+^CD25^+^ Treg Cell Immunosuppression Assay

Mixed lymphocytes reaction (MLR) was used to assess the suppressive activity of CD4^+^ CD25^+^ Treg cells as previously described (14, 15). Briefly, CD4^+^CD8^−^CD25^+^ and CD4^+^CD8^−^CD25^−^ T cells with ratios ranged from 1:1 to 1:16 were cultured for 72 h in 96-well flat-bottomed plates with anti-CD3 (5 μg/ml) and irradiated splenocytes (APCs). After 3 days in culture, ^3^H-thymidine was added to each well for an additional 18 h. ^3^H-thymidine incorporation was measured on a β-scintillation counter (15).

### MLR in Mixed Chimeras

MLRs in chimeras were performed as described previously (12, 16). Briefly, 4 × 10^5^ responder splenocytes were incubated in triplicate with 4 × 10^5^ irradiated (30 Gy) stimulator cells of either B6 (recipient), BALB/c (donor), or C3H (third party) origin or with medium only. After 72 h incubation, cells were pulsed with [3H]-thymidine (Amersham Biosciences, Little Chalfont, Bucks, UK) for 18 h. Incorporated radioactivity was measured using scintillation fluid in a β counter. Stimulation indices were calculated in relation to medium controls.

### Isolation of Lamina Propria Lymphocytes in the Small Intestine

Isolation of lamina propria cells from the small intestine was performed as previously described (17). The whole transplanted small intestine was cut into pieces 0.5 cm in length and shaken twice at 250 rpm for 30 min at 37°C in Hanks’ Balanced Salt Solution (Life Technologies) supplemented with 5% (v/v) fetal bovine serum (CellGro) containing 2 mM EDTA. The remaining intestinal tissues were washed and shaken for 30 min at 37°C in RPMI 1640 plus 5% (v/v) fetal bovine serum and type IV collagenase (1 mg/ml; Sigma). Cell suspensions were enriched by centrifugation at room temperature at 500 *g* for 20 min in 40%/70% Percoll (GE Healthcare) in RPMI 1640. The interface layer cells were used for further analysis.

### Flow Cytometry, Monoclonal Antibodies, and Reagents

Peripheral blood was collected, the red cells were lysed, and the remaining cells washed with a whole blood lysis kit (R&D Systems, Minneapolis, MN, USA). Peripheral blood leukocytes were stained with fluorochrome-conjugated anti-CD3, anti-CD11b, anti-GR1, anti-B220, anti-H-2Kb, anti-Vb11, anti-H-2Kd, anti-Vb8.1/8.2, anti-Vb5.1/5.2 (PharMingen, San Diego, CA, USA), anti-CD4, anti-CD8 (Caltag, Burlingame, CA, USA), or immunoglobulin isotype controls (PharMingen, Caltag). Donor chimerism was expressed as a percentage that was calculated using the following formula: (H-2K^d+^ cells/total gated cells) × 100 (11).

The following monoclonal antibodies (mAbs) were purchased from BD Biosciences PharMingen: anti-mCD4-APC-CY7, anti-mCD45RB-PE, anti-mCD44-PE, anti-mCD62L-PE, anti-mCTLA4-PE, anti-mGITR-PE. Anti-mCD4-FITC, anti-mCD8-PE, anti-mCD4-APC, anti-mFoxp3-PE, and anti-mCD25-PE-CY5 were purchased from eBioscience.

### ELISA for Intestinal Inflammatory Cytokines

Small intestine cytokines were measured with a mouse-specific cytokines ELISA kit (eBioscience, San Diego, CA, USA). Tissues were homogenized in ice-cold RIPA buffer [150 mM NaCl, 50 mM Tris–HCl, 1 mM EDTA, and 1% Triton-X (pH 7.4)], and samples processed for mouse-specific ELISA kits.

### Statistical Analysis

Survival data were analyzed using the Kaplan–Meier method with the log-rank test to verify the significance of the difference in survival between the groups. Data are presented as mean ± SD. Student’s unpaired *t*-test for comparison of means was used to compare groups. *P* < 0.05 was considered to be of significant difference.

## Results

### Expansion of Fresh and Donor-Specific CD4^+^CD25^+^ Treg Cells

**Figure 1:**
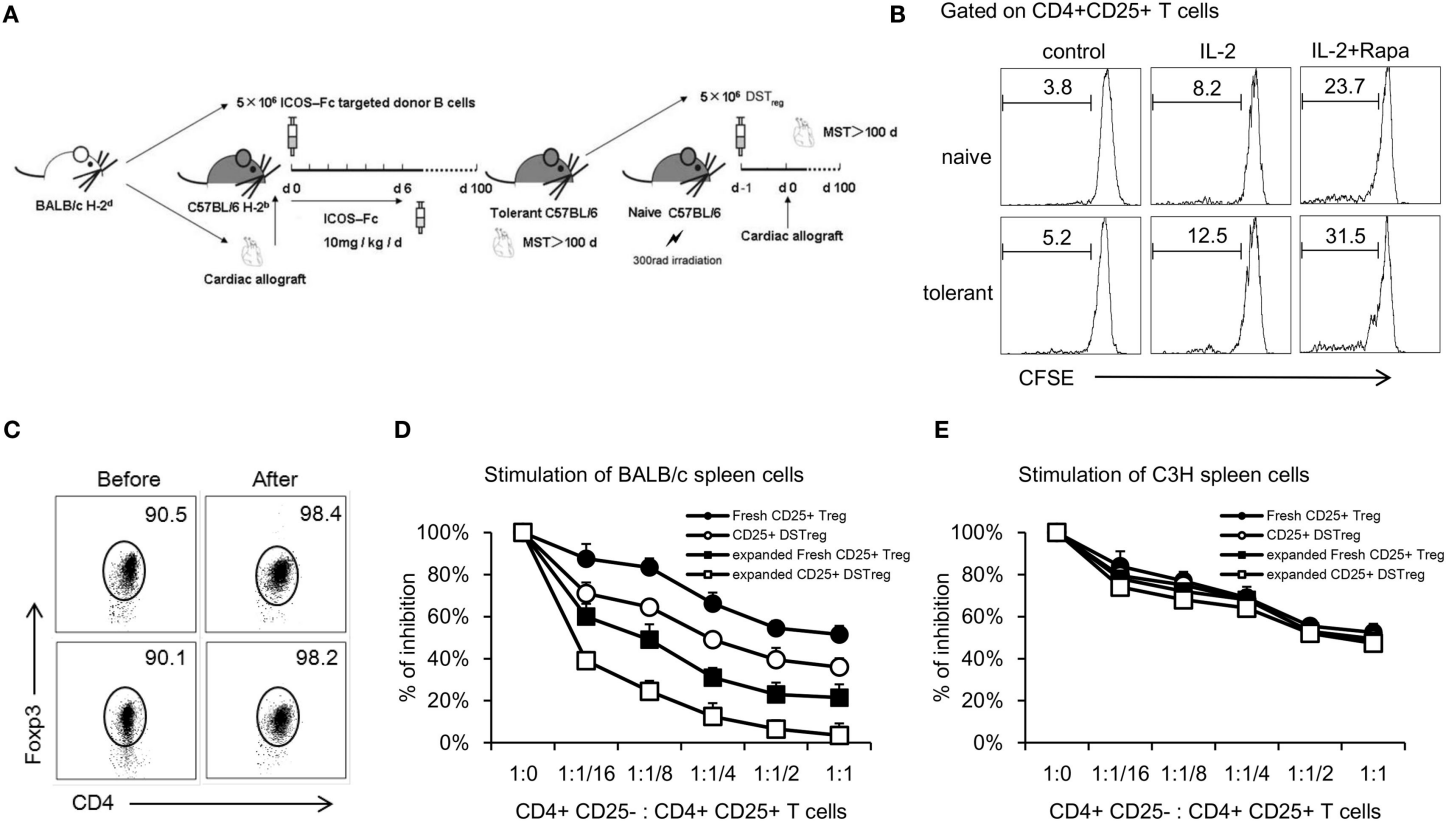
***In vitro* expansion of CD4^+^CD25^+^ Treg cells and comparison with immunosuppressive function of expanded Treg cells**. **(A)** Schematic drawing of the protocol to acquire DSTreg cells from tolerant mice bearing heart allografts. **(B)** Fresh CD4^+^CD25^+^ T cells and DSTreg cells (5 × 10^4^) were separated by magnetic bead sorting and cultivated in the presence or absence of IL-2 (1000 U/ml), splenic donor APCs (10^5^ cells), and rapamycin (100 nM), or in combination for 7 days at 37°C in 5% CO_2_. DSTreg cells proliferated more efficiently in response to the indicated stimulation compared with fresh CD4^+^CD25^+^ T cells as determined by CFSE-labeled cell proliferation assay *in vitro*. **(C)** Representative FACS blot depicting Foxp3 expression among CD4^+^ T cells from naïve or tolerant mice after *in vitro* cultivation. **(D)** Fresh CD4^+^CD25^+^ T cells, *ex vivo*-expanded fresh CD4^+^CD25^+^ T cells, DSTreg cells from tolerant mice, and *ex vivo*-expanded DSTreg cells were assayed for suppressive activity in response to BALB/c spleen cells and third-party spleen cells (C3H) **(E)**. All curves are expressed as percentage of control MLR and represent the combined average values of five individual experiments. Data are shown as mean ± SD (*n* = 3–5 mice/group). One representative of at least two independent experiments is shown. *P < 0.05, **P < 0.01, ***P < 0.005 compared among the indicated groups.

Previously, we have shown that DSTreg cells from tolerant B6 mice bearing cardiac grafts induce cardiac graft acceptance in irradiated B6 mice (Figure 1A) (10). To expand CD4^+^CD25^+^ Treg cells, we first purified single splenocytes from naïve or tolerant B6 mice using a CD4^+^CD25^+^ Regulatory T-cell Isolation Kit (MiltenyiBiotec) (18). The purity of separated cells was confirmed to be >90% (Figure 1C). Numerous *in vitro* and *in vivo* studies have suggested the critical role of rapamycin in expanding naturally occurring CD4^+^CD25^+^Foxp3^+^ Treg cells that are normally found in the naïve splenic CD4^+^ T-cell compartment, as well as in maintaining their suppressive function *in vitro* (19–21). We used similar culture conditions with some modification. After 14 days stimulation, we found that DSTreg and fresh Treg cells could be expanded stably *in vitro*, and the combination of rapamycin plus IL-2 resulted in greatest expansion of fresh CD4^+^CD25^+^ Treg cells (23.7 ± 1.8-fold) and DSTreg cells (31.5 ± 2.1-fold) (Figure 1B). The purity of the expanded cells was >95% (Figure 1C). Furthermore, DSTreg cells proliferated more efficiently than fresh Treg cells in response to IL-2 and rapamycin (Figure 1B). When analyzing the expression of surface markers by flow cytometry, DSTreg cells from tolerant mice expressed equal levels of CD62L, CD44, CTLA-4, and GITR as fresh Treg cells derived from naïve mice did (data not shown). *Ex vivo* fresh Treg and DSTreg cells were also assessed for suppression in MLR assays. Expanded DSTreg cells displayed a more powerful inhibitory function than fresh Treg, expanded fresh Treg, and DSTreg cells (Figure 1D). We also confirmed that the enhanced suppressive function of expanded DSTreg cells was donor specific (Figure 1E). Therefore, we established a method that could expand DSTreg cells *in vitro*.

### Donor-Specific Treg Cell Treatment Leads to Multilineage Mixed Chimerism

**Figure 2:**
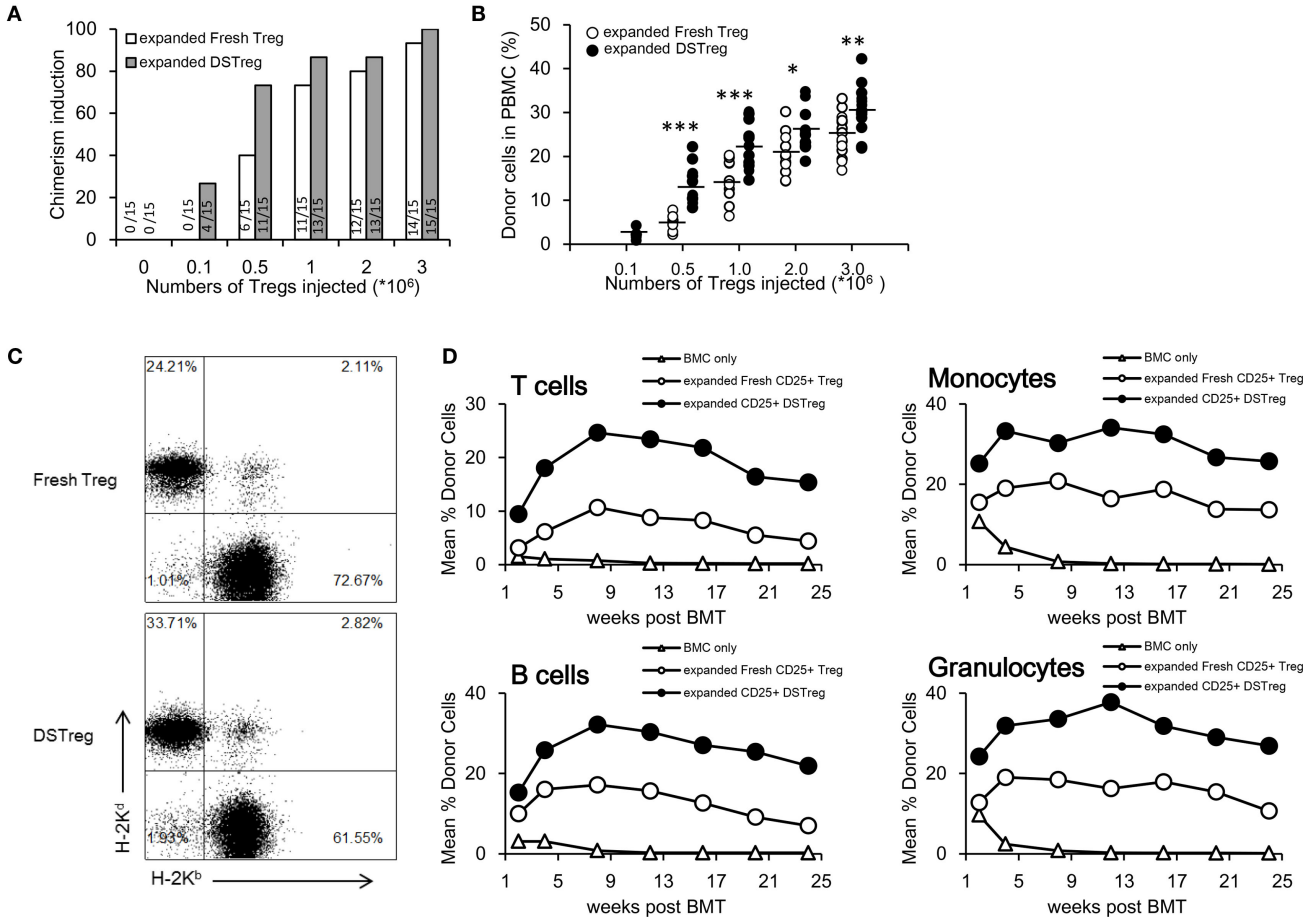
**DSTreg cell treatment together with low-dose irradiation leads to multilineage mixed chimerism**. **(A)** Groups of C57BL/6J mice were transplantated with fully mismatched BALB/c BM cells (2 × 10^7^) under low doses of irradiation (3 Gy total body irradiation at day −1), co-stimulation blockade with abatacept (CTLA-4-Ig) (0.05 mg at day 2), and three doses of rapamycin (0.1 mg at days 1, 0, and 2) were additionally treated with or without different numbers of expanded DSTreg cells, or expanded fresh Treg cells at day 0. Percentages of successfully induced chimeras are shown. Chimerism was considered to be established if donor cells were detectable by flow cytometry within both the myeloid lineage and at least one lymphoid lineage for the duration of follow-up. **(B)** Hematopoietic reconstitution was assessed at 3 weeks after BMT. Values for individual mice are shown; bars indicated means. **(C)** Typical FACS plots of H-2K^b^ (recipient) versus H-2K^d^ (donor) staining were carried out 3 weeks after BMT. **(D)** Donor (H-2D^d^) chimerism among leukocytes was assessed by flow cytometry of peripheral blood at multiple time points (2, 4, 8, 12, 16, 20, and 24 weeks post-BMT) and is shown as mean percentage. *P < 0.05, **P < 0.01, ***P < 0.005 compared among the indicated groups.

Next, we investigated the potency of expanded DSTreg and expanded fresh Treg cells for induction of chimerism and tolerance. Under the cover of co-stimulation blockade with abatacept, a low dose of 3 Gy irradiation and three doses of rapamycin, B6 mice received a conventional dose of fully mismatched BALB/c BM (2 × 10^7^ cells per mouse), together with different numbers of expanded DSTreg or fresh Treg cells (10^5^, 5 × 10^5^, 10^6^, 2 × 10^6^, or 3 × 10^6^ per mouse). Most recipients treated with Treg cells developed mixed chimerism, whereas no chimerism was detected without Treg cell treatment 4 weeks after BMT (Figure 2A). BMT recipients treated with expanded DSTreg cells displayed higher chimerism rates than those with expanded fresh Treg cells, and cell numbers could even be reduced to 5 × 10^5^ cells/mouse (Figure 2A). Chimerism levels in recipients treated with expanded DSTreg cells were also significantly higher than those in recipients treated with expanded fresh Treg cells (Figures 2B,C). The best induction of stable mixed chimerism was achieved with injection of 3 × 10^6^ cells/mouse (Figure 2C); therefore, we used this number of cells for subsequent experiments. We also discovered that BMT recipients treated with expanded DSTreg cells achieved substantial levels of T cells, B cells, granulocytes, and macrophages chimerism at indicated time points post-BMT, while the levels of chimerism in recipients treated with expanded fresh Treg cells were relatively low (Figure 2D). Therefore, these results suggest that expanded DSTreg cells were more potent than expanded fresh Treg cells in the induction of hematopoietic chimerism.

### Clinical Manifestation and Graft Survival Rates

**Figure 3:**
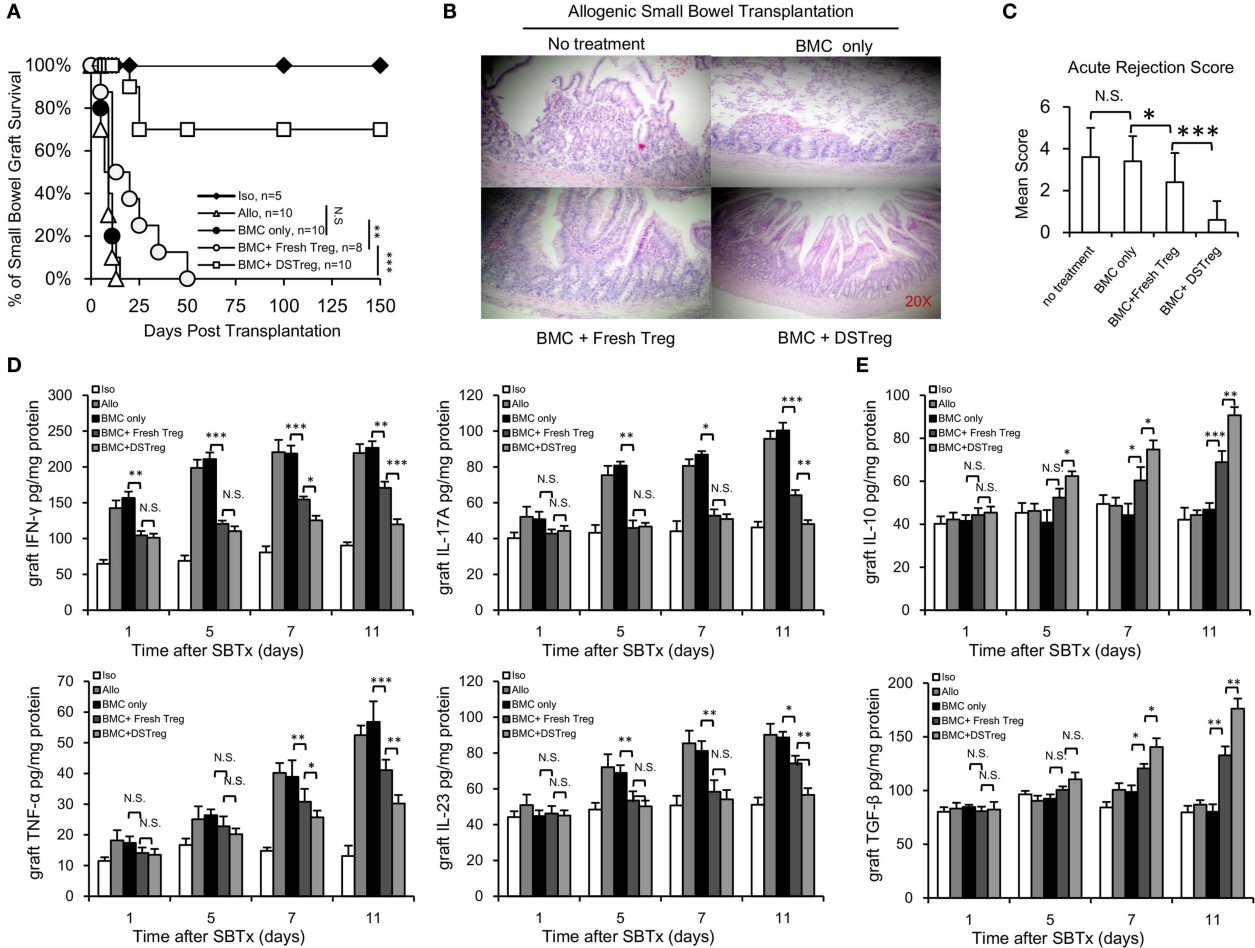
**Chimeras induced by DSTreg cells developed full donor-specific intestine allograft tolerance, and tolerant chimeras displayed hypo-inflammatory responses in the intestinal allografts**. **(A)** Graft survival of intestinal allografts. **(B)** Representative hematoxylin-and-eosin-stained (original magnification: ×100) sections of intestinal allografts from recipients treated with low-dose irradiation (3 Gy) and BM cells, with or without expanded DSTreg cells, and expanded fresh Treg cells (14 days post-SBT). **(C)** Allografts were assigned an acute rejection score by a blinded pathologist. **(D,E)** Cytokine concentrations in the recipient intestinal allografts were measured by ELISA on days 1, 5, 7, and 11 after SBT. Data are shown as mean ± SD (*n* = 4–7). One representative of at least two independent experiments is shown. *P < 0.05, **P < 0.01, ***P < 0.005 compared among the indicated groups.

After the establishment of BM chimerism, we further investigated whether mixed chimerism induced by Treg cells could promote intestinal allograft tolerance. SBT was performed 4 weeks after BMT. The intestinal grafts in the allogeneic group were all rejected within 14 days. Intestinal grafts in recipients that received BM cell infusion showed similar survival rates to those in the allogeneic group (Figure 3A), while intestinal grafts in the BM cells + expanded fresh Treg cells group survived for an extended period of 50 days after transplantation (Figure 3A). Intestinal grafts in the chimera recipients induced by expanded DSTreg cells survived for the longest period, and more than half of the grafts survived for the duration of follow-up, without the presence of both acute and chronic rejection (Figure 3A). When comparing the histological results among each group, chimeras induced through DSTreg cell infusion demonstrated viable and best-preserved structure of mucosa and villi (Figure 3B). The stoma in this group also had good structure. The relative rejection score was also significantly lower in chimeras induced by BM and DSTreg cell treatment (Figure 3C).

The main factors controlling organ rejection are the balance between cellular immune responses mediated by T-helper cells that produce numerous proinflammatory cytokines and inhibitory cytokines. Thus, the grafts concentrations of inflammatory-response-related Th1/Th2 and Th17/Treg cytokines were also assayed with ELISA. The concentrations of tumor necrosis factor (TNF)-α, IL-17A, IL-23, and interferon (IFN)-γ all increased gradually in the allogeneic control group (Figure 3D). Compared to the allogeneic control group, the concentrations of IL-23, IFN-γ, and IL-17A in the grafts of chimeras were significantly lower on days 5, 7, and 11 than those in the allogeneic control group (Figure 3D). The concentrations of inhibitory cytokines transforming growth factor-β and IL-10 were significantly higher in the chimera group than in the allogeneic group on days 5, 7, and 11 (Figure 3E), and the levels of these two inhibitory cytokines were higher in the BM + DSTreg cell group than in the BMC + fresh Treg cells group (Figure 3E). These results suggest that recipients treated with BMC + DSTreg cells can better accept small bowel allografts, with low levels of inflammatory response and acute rejection.

### Increased Infiltration of CD4^+^Foxp3^+^ Treg Cells in Small Bowel Grafts and Deletion of Donor-Reactive T Cells of Mixed Chimeras Induced by BM and DSTreg Cell Infusion

**Figure 4:**
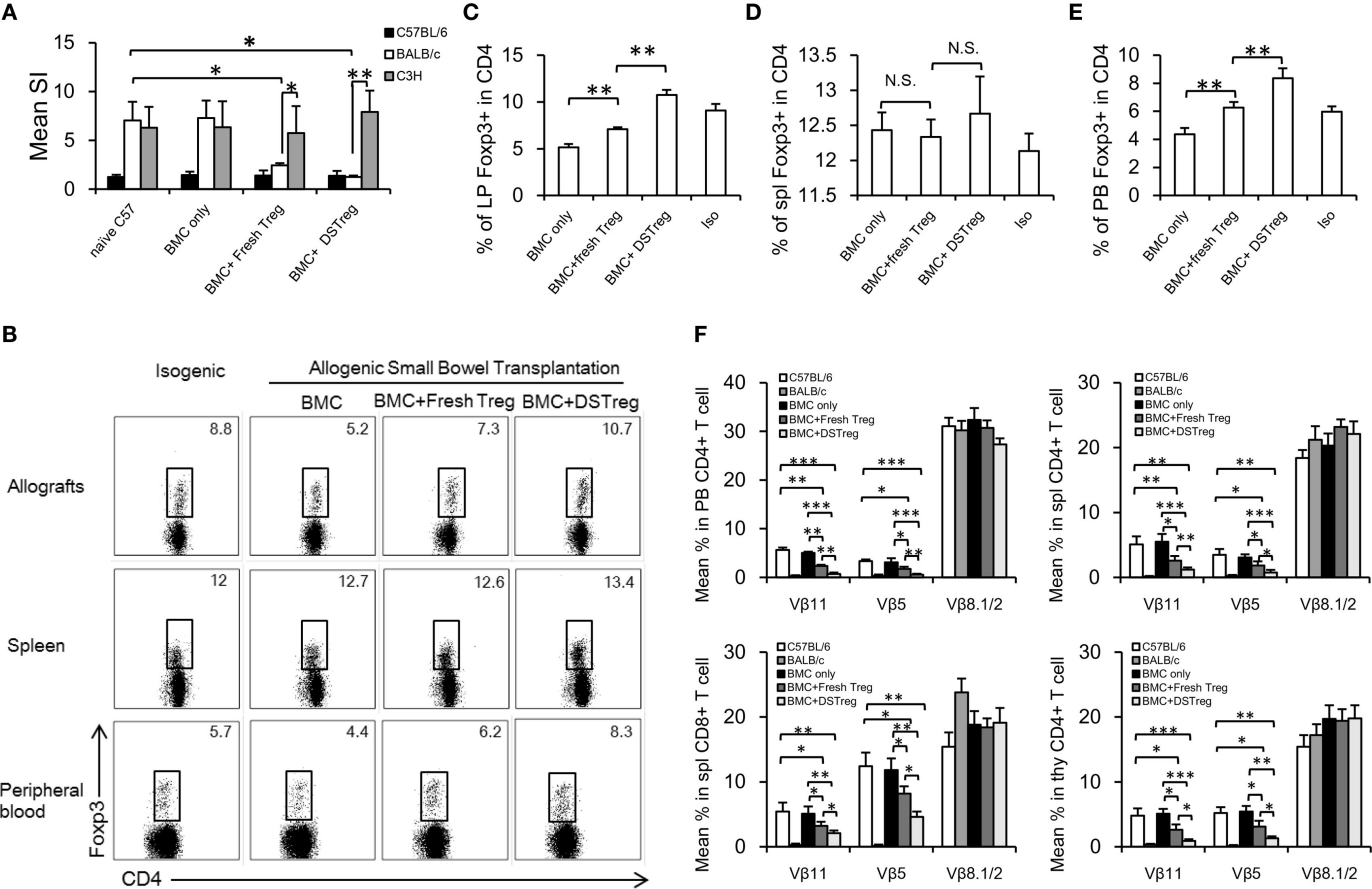
**Regulatory mechanism and clonal deletion contribute to intestinal allograft acceptance in chimeras treated with expanded DSTreg cells**. **(A)** Donor reactivity was assessed in MLRs at 8 weeks post-BMT. Donor-specific responses and third-party reactivity were measured in each group. Simulation indices were calculated by dividing the mean cpm from responses against recipient (C57BL/6J), donor (BALB/c), or third-party (C3H) stimulator cells by mean background cpm (i.e., cpm with no stimulator population). The percentage of CD4^+^Foxp3^+^ cells in recipient allografts **(B,C)**, spleen **(D)**, and peripheral blood **(E)** was measured by multicolor flow cytometry on day 14 after SBT. **(F)** Deletion of donor-reactive T cells in chimeras was shown by assessing percentages of Vβ11, Vβ5, and Vβ8. Multicolor flow cytometry was used for measurement in selected mice at 8 weeks post-BMT. Chimeras treated with expanded DSTreg cells showed significant peripheral and central clonal deletion among donor-reactive T cells, as measured by percentage of Vβ11 and Vβ5 (but not Vβ8). Data are shown as mean ± SD (*n* = 4–6). One representative of at least three independent experiments is shown. *P < 0.05, **P < 0.01, ***P < 0.005 compared among the indicated groups.

To assess tolerance in these fresh Treg and DSTreg induced chimeras, *in vitro* MLR assays were performed to evaluate self-reactivity, donor-reactivity, and third-party reactivity. In chimeras treated with Treg cells, responsiveness toward the donor was almost reduced to the level of self-reactivity (Figure 4A), whereas third-party reactivity was preserved (Figure 4A). CD4^+^Foxp3^+^ Treg cells have been implicated to play a crucial role in homeostasis of intestinal immunity (18, 22, 23), and dysregulation of the number and function of Treg cells contributes to the development of intestinal transplantation rejection (24, 25). Previous results have also suggested higher levels of inhibitory cytokines relative to Treg cells in recipients treated with expanded DSTreg cells. Therefore, we obtained intestinal grafts and directly assessed the infiltration of CD4^+^Foxp3^+^ Treg cells as previously described (17). The percentage of infiltrating CD4^+^Foxp3^+^ Treg cells was significantly higher in grafts of chimeras treated with BM + expanded DSTreg cells compared to those of chimeras treated with BM cells only or BM + expanded fresh Treg cells (Figures 4B,C). Although the percentage of Foxp3^+^ Treg cells in CD4^+^ T cells in the spleen of recipients treated with expanded DSTreg cells was higher than that in recipients treated with expanded fresh Treg cells, no significant difference was discovered (Figure 4D). The frequency of CD4^+^Foxp3^+^ Treg cells in peripheral blood is negatively correlated with severity of graft versus host diseases in humans (26) and is also regarded as a biomarker for hematopoietic cell transplantation outcomes (27). Similar results were also obtained in our model (Figure 4E).

Mixed chimerism was induced in our model and chimeric recipients displayed hyporesponsiveness to donor antigens; therefore, deletion of intrathymic and peripheral donor-reactive T cells might also underlie the lack of responsiveness to donor antigens. To test this hypothesis, we examined the levels of certain Vβ subunits within the T-cell receptor repertories. The frequencies of Vβ11^+^ and Vβ5^+^ peripheral CD4^+^ T cells were low in chimeras treated with expanded DSTreg cells, which suggested the establishment of peripheral clonal deletion, while it was incomplete in chimeras with BM cells, low-dose irradiation, and expanded fresh Treg cells (Figure 4F). No deletion was seen in recipients without Treg cell treatment (Figure 4F). Furthermore, significant intrathymic deletion was also achieved in recipients treated with expanded DSTreg cells (Figure 4F).

### Donor-Specific Skin Graft Acceptance in Mixed Chimeras Receiving BM and DSTreg Cell Infusion, and Maintenance of Normal Immune Response to Third-Party Grafts

**Figure 5:**
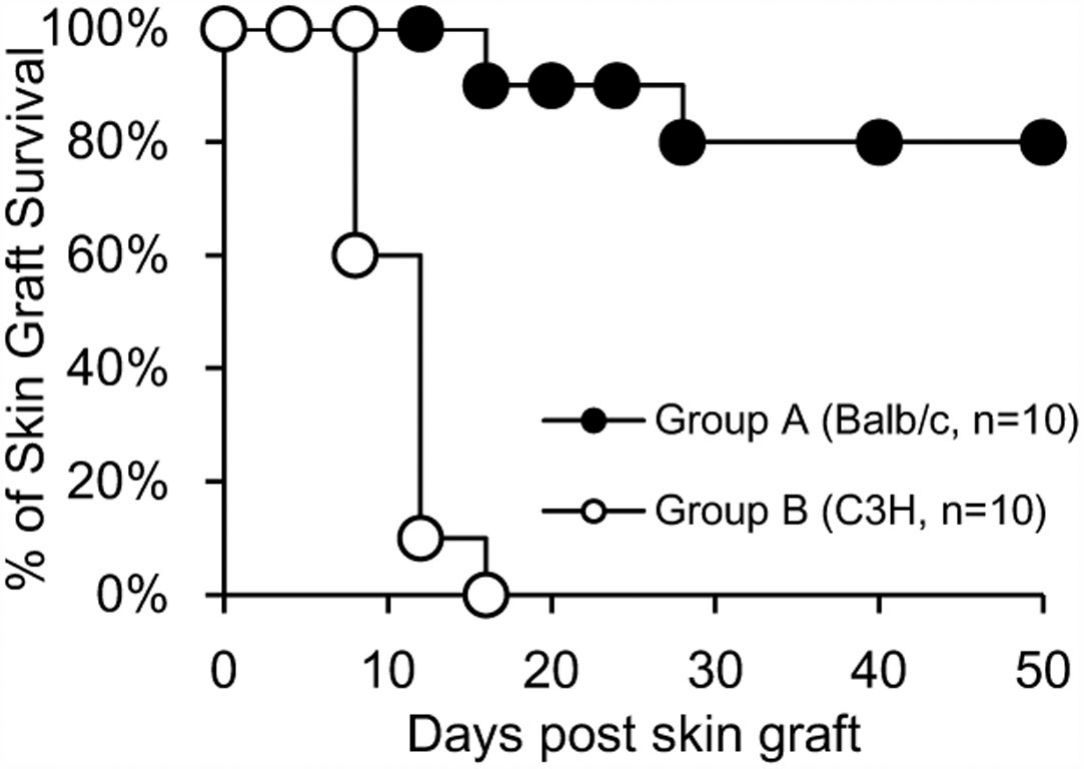
**Mixed chimeras of DSTreg cell infusion group received donor-specific skin grafts and retained normal immune response to third-party grafts**. Skin transplantation was performed 100 days after SBT in recipients (C57BL/6) treated with DSTreg cells, and survival curve of skin grafts from donor-specific Balb/c mice and C3H mice was shown. Donor-specific Balb/c skin grafts survived in most chimeras, whereas C3H skin was rejected. *P < 0.05, **P < 0.01, ***P < 0.005 compared among the indicated groups.

To determine whether or not tolerance was donor-specific in recipients treated with DSTreg cells after SBT, skin transplantation was performed 100 days after SBT. Mice receiving DSTreg cells that did not accept small bowel grafts rejected both BALB/c and C3H skin grafts within 14 days, while mice achieving small bowel grafts permanently also accepted BALB/c skin grafts permanently. These mice also rejected C3H skin grafts, which further showed that mice that accepted small bowel grafts achieved donor-specific transplantation tolerance (Figure 5).

## Discussion

We (28) and others (23, 29) have suggested the critical role of Treg cells in maintaining intestinal homeostasis, and better control of the number and function of Treg cells in GALT, especially in the intestine, might be a promising prospect for the acceptance of intestinal allografts. How to maintain long-term DSTreg cells and/or expand them *in vivo* has always been a central problem in immune tolerance, and isolating sufficient numbers of Treg cells for *in vivo* use is also a significant clinical challenge (21). Previously, we have shown that donor-specific transfusion with complete blockade of ICOS/B7h signaling can achieve immune tolerance (10). Furthermore, this allograft acceptance is transferable and mediated by CD4^+^CD25^+^Foxp3^+^ T cells from recipient mice. To acquire large numbers of these DSTreg cells for further use, we tried to expand the cells *in vitro*. Mature DCs have been suggested to be the most potent APCs to expand antigen-specific Treg cells in the presence of high-dose IL-2 (30). Since spleen cells are a mixture of many cell types with only 1–1.5% being DCs, among which the majority are in an immature state, we used BMDCs as donor-specific APCs to stimulate Treg cells, with or without rapamycin or IL-2, alone or in combination. After 2 weeks of co-culture, our results clearly showed that BMDCs could expand these DSTreg cells with potent suppressive function *in vitro* in the presence of IL-2 and rapamycin, which is consistent with other *in vitro* and *in vivo* results (19–21). *In vitro* results also demonstrated that these expanded DSTreg cells displayed more powerful immunosuppressive function. However, it has been shown that infusion of DSTreg cells alone only delays CD4^+^ T-cell-mediated skin graft rejection and CD8^+^ T-cell-mediated allograft rejection (11, 31, 32), and results from highly immunogenic organ transplantation models are still frustrating (11). We (4) also acquired similar results in SBT, which drove us to seek better strategies for immune tolerance in SBT. Studies have also suggested that rodent models for tolerance through mixed chimerism are among the most robust, which might be the best candidate for clinical trials (8, 33, 34). Recently, long-term stable kidney allograft survival without maintenance immunosuppression was also achieved by infusion of BM cells (35). Therefore, chimerism might be a promising strategy for intestinal transplantation.

Various mixed chimerism protocols have been developed including the use of immunosuppressive drugs, co-stimulation blockade (36, 37), Foxp3^+^ Treg cell application (8, 38), and T-cell depletion (11, 39). Among all these, strategies based on the use of co-stimulation blockade are the most potent at inducing mixed chimerism (6). However, there are few reports on protocols for promoting stable intestinal allograft acceptance. Guo et al. have conducted a series of studies on biological agents that delay intestinal acute rejection and found that chimerism with anti-CD40L mAb, CTLA4-Ig, donor BM, and busulfan prolong intestinal allograft survival (11). However, their strategy still failed to achieve long-term survival of intestinal allografts with different levels of chimerism and persistence of donor-reactive T cells in recipients. Furthermore, they could not identify Treg cells in chimeric recipients bearing intestinal allografts, which suggest the absence of a regulatory mechanism in this model. Recently, combining Treg therapy with non-cytoreductive BMT has been suggested to promote acceptance of heart grafts in mice (12). Although this kind of Treg-cell-induced strategy achieved immune tolerance, the levels of chimerism observed were low and depletion of donor-reactive T cells was also incomplete, which might not be suitable for application to immunogenic organs like the intestine (8, 12). Furthermore, in addition to tolerization of intrathymic newly developing T-cell repertoire, pre-existing mature donor-reactive T cells also need to be tolerized through peripheral mechanisms in such protocols (9).

In our study, we found that DSTreg cells from tolerant mice stably induced mixed chimerism and further promoted the intestinal allograft acceptance. The percentage of Treg cells in the intestine was also significantly high in the DSTreg-cell-induced chimeras, which might have contributed to the low levels of inflammatory response. Similar results were found in peripheral blood, which strongly suggests achievement of immune tolerance in these recipient mice. Besides, clonal deletion is considered the backbone of tolerance through chimerism (40), and most groups investigating tolerance associated with chimerism-inducing strategies have reported deletion of donor-reactive CD4^+^ T cells (11). We also observed significant deletion of Vβ5^+^ and Vβ11^+^ MMTV-reactive CD4^+^ and CD8^+^ T cells in DSTreg-cell-induced chimeras. Therefore, clonal deletion and regulatory mechanisms both contribute to promote allograft acceptance in our study. We also further demonstrated that immune tolerance induced in our model was donor specific.

In conclusion, we have developed a new mixed-chimerism-inducing protocol using the DSTreg cells acquired from tolerant mice bearing heart allografts and have promoted the acceptance of intestinal allografts. These results underlie the clinical potential of Treg-cell-based chimerism and subsequent prevention of solid organ transplantation rejection that is highly immunogenic. Further studies on determining the origin (donor or recipient) and migration pattern of Treg cells in these tolerant mice bearing intestinal allografts are needed so that they can be used clinically.

## Author Contributions

J-FD and W-XG conceived and designed the experiments; X-FS and J-PJ performed the experiments; J-JY and W-ZW analyzed the data; J-PJ, X-FS, and J-JY contributed reagents/materials/analysis tools; J-FD and X-FS wrote the paper.

## Conflict of Interest Statement

The authors declare that the research was conducted in the absence of any commercial or financial relationships that could be construed as a potential conflict of interest.

## Abbreviations

(DS) Treg, (donor-specific) regulatory T cells; APC, antigen-presenting cell; BMC, bone marrow cells; BMDCs, bone marrow-derived dendritic cells; CFSE, carboxyflurescein diacetate succinmidyl ester; CTLA-4, cytotoxic T lymphocyte antigen-4; GM-CSF, granulocyte-macrophage colony-stimulating factor; ICOS, inducible co-stimulator; IFN-γ, interferon-γ; IL, interleukin; MLR, mixed lymphocytes reaction; SBT, small bowel transplantation; TNF-α, tumor necrosis factor-α.
